# Diagnosis: Infantile Malign Osteopetrosis

**DOI:** 10.4274/tjh.2013.0405

**Published:** 2014-09-05

**Authors:** Sevgin Taner, Ali Fettah, Neşe Yaralı, Sevde Seçer, Özge Ağlamış, Bahattin Tunç

**Affiliations:** 1 Ankara Children’s Hematology and Oncology Research Hospital, Clinic of Pediatric Hematology, Ankara, Turkey

**Keywords:** Hypocalcemic seizure, Infantile malign osteopetrosis, TCIRG1 gene mutation

## QUIZ IN HEMATOLOGY

**Figure 1:**
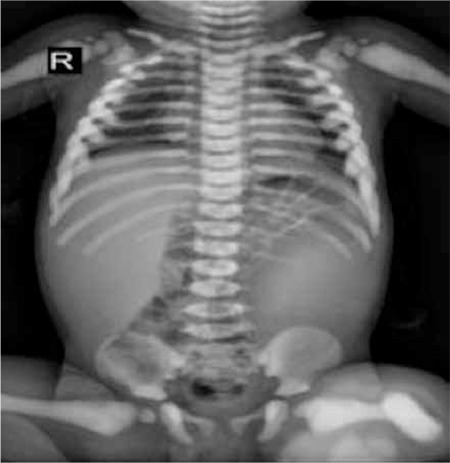
Posteroanterior chest X-ray revealed a uniform increase in bone density.

**Figure 2:**
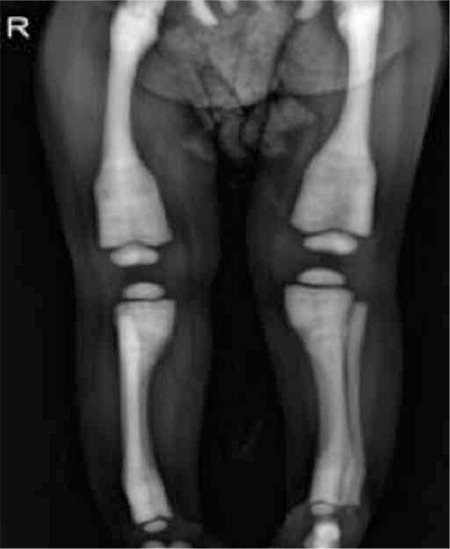
Radiograph of limbs shows Erlenmeyer flask deformity of distal femur and generalized increased bone density with the obliteration of the marrow cavity.

**Figure 3:**
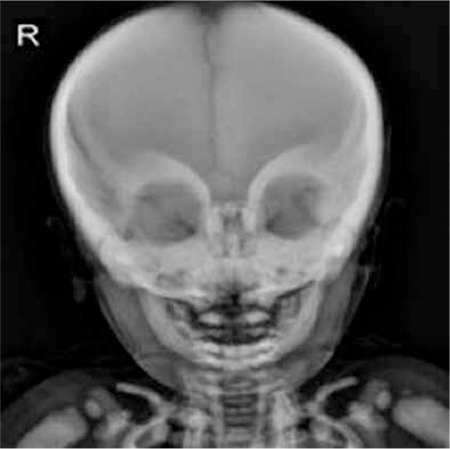
Radiograph of skull showing sclerosis and thickening of orbital rims.

A 2-year-old patient was admitted to the hospital on the fifth day of his life with hypocalcemic seizure. When he was 3 months of age, blindness and hepatosplenomegaly were noticed. His leukocyte count was 36.8x10³/µL, hemoglobin level was 8 g/dL, and platelet level was 103x10³/µL; he was referred with suspicion of infantile leukemia. His peripheral smear demonstrated several immature myeloid cells and normoblasts, while no blastic cells were observed. Chest radiograph showed a generalized increase in bone density (Figure 1). Radiographs of the skull and limbs showed generalized increase in bone density (Figures 2 and 3).

## DIAGNOSIS

The patient was diagnosed with osteopetrosis, which was confirmed due to mutation in the TCIRG1 gene [g.11279G>A(IVS18+1) paternal allele, g.8280_9560del (ex. 11-12-12), p.Ala389AspfsX151 maternal allele].

Osteopetrosis originates from reduced or complete lack of osteoclast function and, as a consequence, impairment of bone resorption [1]. At least 10 genes have been identified as causative in humans [2]. Osteopetrosis varies in its presentation and severity. The autosomal recessive form is the most severe form, with life-threatening complications such as bone marrow failure; it is usually diagnosed before 1 year of age and can mimic leukemia [1,3]. The bone seems to be the only affected tissue and the defect can be almost completely reversed by hematopoietic stem cell transplantation [1,3].
